# Thyroid hormone sensitivity and insulin resistance in euthyroid adults in Beijing: A cross-sectional study

**DOI:** 10.1097/MD.0000000000049117

**Published:** 2026-06-12

**Authors:** Min Zhang, Aiyun Yu, Weihan Zhang, Jianjun Wang, Lijun Li, Lintao Shi

**Affiliations:** a Department of Special Service Health Management, Ninth Medical Center of Chinese PLA General Hospital, Beijing, China; b Department of Endocrinology, Ninth Medical Center of Chinese PLA General Hospital, Beijing, China; c Department of Dermatology, Ninth Medical Center of Chinese PLA General Hospital, Beijing, China.

**Keywords:** insulin resistance, HOMA-IR, thyroid hormone sensitivity

## Abstract

This study aimed to investigate the relationship between indices of thyroid hormone sensitivity and insulin resistance (IR) in euthyroid adults. In this cross-sectional study of 1393 euthyroid individuals from Beijing, participants were stratified into IR (Homeostatic Model Assessment for Insulin Resistance ≥ 2.5) and non-IR groups. We calculated indices of central thyroid hormone sensitivity: Thyroid Feedback Quantile-based Index, Thyrotroph T4 Resistance Index, and Thyroid-Stimulating Hormone Index and the peripheral free triiodothyronine/free thyroxine ratio. Multivariable logistic regression analyses were performed to determine their associations with IR, adjusting for key metabolic confounders including age, sex, body mass index, blood pressure, glycated hemoglobin, and lipids. A significant inverse association was observed between central thyroid hormone resistance and IR. After full adjustment, participants in the highest quartile of Thyroid Feedback Quantile-based Index, Thyrotroph T4 Resistance Index, and Thyroid-Stimulating Hormone Index had approximately 50% lower odds of IR. In contrast, a higher free triiodothyronine/free thyroxine ratio, indicating enhanced peripheral conversion, was positively associated with increased odds of IR. These relationships followed a threshold effect, being most pronounced in the extreme quartiles. Among euthyroid adults, reduced central sensitivity to thyroid hormones is independently associated with lower odds of IR, while increased peripheral conversion is linked to higher odds. This dissociation underscores the critical role of the hypothalamic–pituitary–thyroid axis set-point and peripheral hormone activation in metabolic health, suggesting these indices could be valuable for stratifying metabolic risk.

## 1. Introduction

Insulin resistance (IR) is defined as a condition in which the tissues of the body fail to respond normally to insulin.^[1]^ Obesity, hypertension, atherosclerotic cardiovascular disease (CVD), and dyslipidemia have been shown to be associated with IR and hyperinsulinemia.^[2–4]^ In clinical practice, early detection and intervention of IR may prevent diseases related to IR.

Thyroid hormones (THs) exert profound effects on the cardiovascular system, influencing heart rate, contractility, vascular tone, lipid metabolism, and endothelial function.^[5–7]^ Overt and hyperthyroidism are well-established risk factors for accelerated atherosclerosis and CVD.^[8,9]^ Recent evidence suggests that even when thyroid hormone levels are within the normal range, subtle variations in thyroid hormone levels are associated with cardiovascular risk factors and outcomes, including dyslipidemia, hypertension, metabolic syndrome, and incident CVD events.^[10]^

THs play a crucial role in the process of metabolic regulation. Novel indices derived from standard thyroid function tests (TSH, free thyroxine [FT4]) have been developed and validated as surrogates for central (hypothalamic-pituitary) sensitivity: the Thyroid Feedback Quantile-based Index (TFQI),^[11]^ the Thyrotroph T4 Resistance Index (TT4RI),^[12]^ and the Thyroid-stimulating Hormone Index (TSHI).^[13]^ Previous studies have suggested that peripheral resistance to THs (e.g., due to thyroid hormone receptor mutations causing systemic hormone resistance) may be a risk factor for metabolic diseases. However, emerging evidence indicates that decreased central sensitivity to THs at the hypothalamic-pituitary level as reflected by elevated TFQI, TT4RI, and TSHI indices is paradoxically associated with lower risk of prediabetes, hyperuricemia, and metabolic syndrome.^[10,14,15]^ Yang et al reported that decreased sensitivity of the central thyroid is a risk of developing diabetes. IR is the key mechanism leading to the development of diabetes. Wang et al used the glucose triglyceride (TG) index to represent IR; they found that the reduced sensitivity to THs was associated with a higher level of IR in adults with normal or overweight body weight and normal thyroid function.^[14]^

Previous studies did not use Homeostatic Model Assessment for Insulin Resistance (HOMA-IR) to investigate the relationship between thyroid hormone sensitivity and IR. Therefore, the primary objective of this study was to investigate the relationship between these indices of thyroid hormone sensitivity and HOMA-IR in a cohort of rigorously defined euthyroid individuals.

## 2. Methods

### 2.1. Study design and population

**Figure 1.**
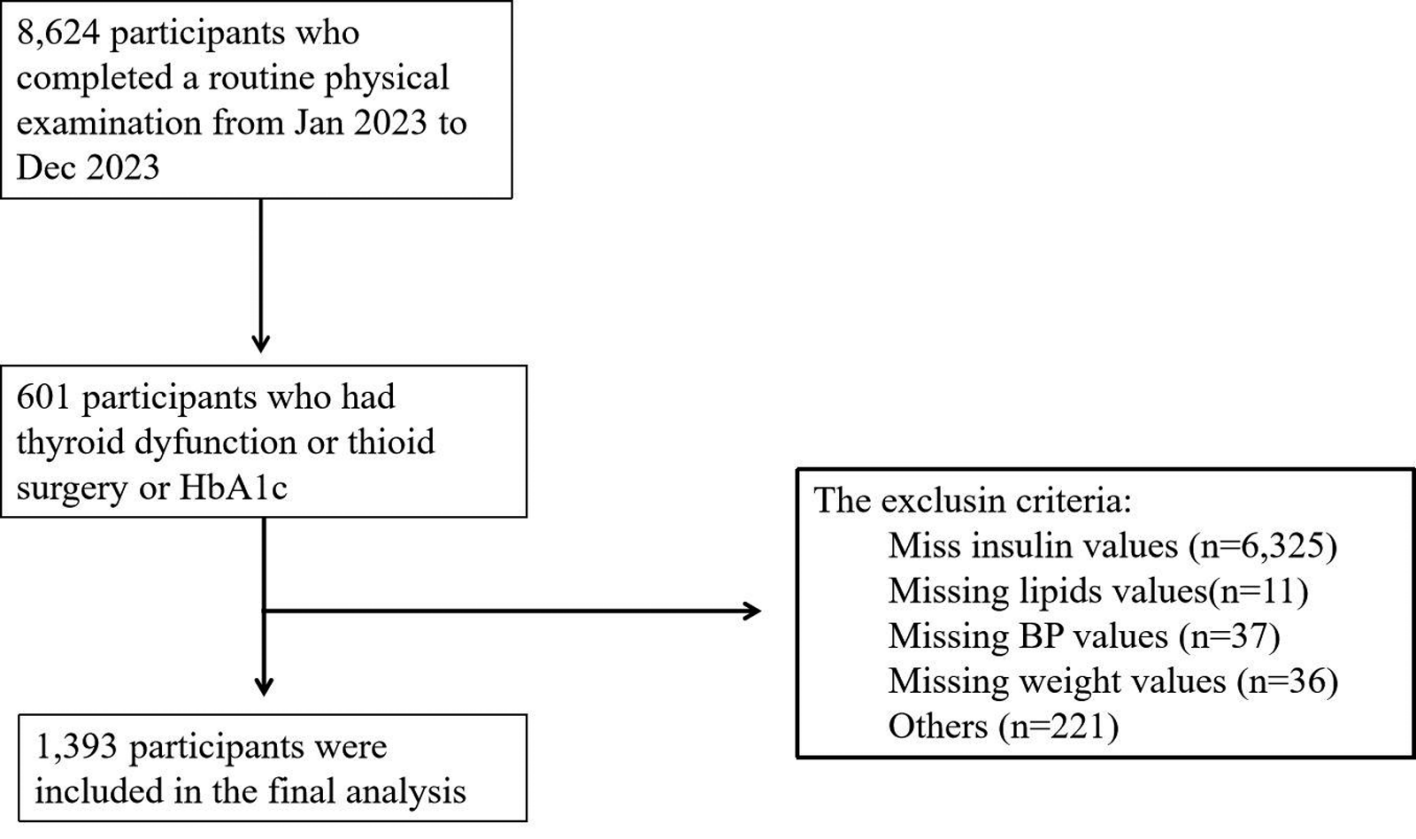
The flowchart of the population. BP = blood pressure, HbA1c = glycated hemoglobin.

This was a cross-sectional study that retrospectively recruited 8624 participants who underwent the annual health checkup in the health examination center of the 9th Medical Center of Chinese PLA General Hospital from January 2023 to January 2024. The inclusion criteria were^[1]^ with normal thyroid function, serum TSH within the laboratory-specific reference range (0.55–4.78 IU/mL), FT4 (0.89–1.76 ng/dL) and free triiodothyronine (FT3; 2.3–4.2 pg/mL). The exclusion criteria were as follows: incomplete data, age <18 years, with a known history of thyroid disease or thyroid surgery, had been treated with drugs potentially altering thyroid hormone concentrations such as amiodarone and corticosteroids, with a history of pituitary disease, with a history of malignancy, and pregnancy. Finally, a total of 1393 participants (mean age: 50.8 ± 11.1 years) were enrolled in the current analyses. A flowchart of the participants recruitment process is shown in Figure 1.

This research was conducted with the principles of the Declaration of Helsinki in mind and was approved by the Ethics and Research Committee of the Ninth Medical Center of Chinese PLA General Hospital (approval date: November 12, 2024). Private details were anonymized to secure confidentiality; analyses were conducted with strict security, and the data were only used for scientific purposes. As this was a retrospective study using de-identified data, the need for informed consent was waived by the Ethics and Research Committee.

### 2.2. Clinical and laboratory assessment

Demographics and clinical data: standardized questionnaires and clinical examinations were used to collect data on age, sex, medical history (hypertension, diabetes mellitus, dyslipidemia), and medication use. Height and weight were measured to calculate body mass index (BMI). Blood pressure (BP) was measured using a calibrated sphygmomanometer after 5 minutes of rest. Euthyroid was defined as thyroid-stimulating hormone (TSH; 0.75–5.6 IU/mL), FT4 (12.0–22.0 pmol/L), and FT3 (3.1–6.8 pmol/L) within the reference ranges.

Laboratory measurements: blood samples were drawn from all participants between 7:00 and 9:00 am after 8 to 10 hours of overnight fasting. Blood samples were centrifuged within 30 to 45 minutes of collection. Weight, height, and BP were measured according to standard protocols by the same trained staff. TSH, FT4, and FT3 were evaluated by electro-chemiluminescence immunoassay using an Abbott Architect i2000 (Roche 801, Germany). Serum TG, total cholesterol, high-density lipoprotein cholesterol (HDL-C), and low-density lipoprotein cholesterol were measured using the enzymatic colorimetric method with cholesterol esterase, cholesterol oxidase, and glycerol phosphate oxidase, respectively. All biochemical tests were performed on the day of sampling using commercial kits by the auto-analyzer (Roche 801), and samples were analyzed only when quality control met the acceptable criteria.

### 2.3. Calculations

The formulas for indexes representing IR are as follows: HOMA-IR = (fasting blood glucose [mmol/L] × fasting insulin [µIU/mL]]/22.5).^[15]^

The equations used for calculating thyroid sensitivities are as follows:

TFQI = cdf FT4 − (1 − cdf TSH), cdf: cumulative distribution function^[11]^;
TSHI = ln TSH (mIU/L) + 0.1345 × FT4 (pmol/L)^[13]^;
TT4RI = FT4 (pmol/L) × TSH (mIU/L)^[11]^;
FT3 = FT3/FT4;
BMI = weight (kg) / height (m)².

### 2.4. Statistical analysis

Statistical analyses were performed using SPSS Statistics 26.0 (IBM Corp.) and GraphPad Prism 9.0 (GraphPad Software). Continuous variables are presented as mean ± standard deviation for normally distributed variables or median (interquartile range) for skewed variables, frequencies (proportions). Continuous variables were compared using the Student *t* test or the Mann–Whitney *U* test. Categorical variables were compared using the chi-square test. Logistic regression analysis was performed to determine the relationships between IR and thyroid system markers. The logistic regression analysis yielded odds ratios (OR) and 95% confidence intervals (CI). A two-tailed *P*-value <.05 was deemed statistically significant.

## 3. Results

### 3.1. Baseline characteristics

A total of 1393 euthyroid participants (mean age 50.8 years, 61.1% male) were included, stratified by 560 (40.2 %) subjects who were classified as IR. Grouping was conducted based on the cutoff point of HOMA-IR at 2.5.^[15]^ Those with HOMA-IR ≥2.5 were classified as the IR group, while those with HOMA-IR <2.5 were classified as the non-IR group. Participants with IR were significantly younger with a male predominance (70.7% vs 54.6%, *P* < .001) and presented with a heavier metabolic burden. BMI was 2.3 kg/m^2^ higher in the IR group (26.8 ± 3.3 vs 23.8 ± 3.2, *P* < .001), accompanied by elevations in both systolic and diastolic BP (+7.1 mm Hg and + 4.2 mm Hg, respectively, both *P* < .001). Fasting glucose, glycated hemoglobin (HbA1c), and fasting insulin were all markedly increased (fasting blood glucose: 6.11 ± 1.74 vs 5.32 ± 0.69 mmol/L; HbA1c: 6.10 ± 1.07 vs 5.72 ± 0.58 %; insulin: 16.6 ± 7.6 vs 6.9 ± 2.2 µIU/mL; all *P* < .001). Lipid profile showed higher TGs (median 1.59 vs 1.31 mmol/L, *P* < .001) and low-density lipoprotein-cholesterol (2.80 ± 0.73 vs 2.72 ± 0.76 mmol/L, *P* = .034), whereas HDL-C was lower (1.46 ± 0.33 vs 1.62 ± 0.38 mmol/L, *P* < .001; Table 1).

**Table 1:** Baseline characteristics according to the HOMA-IR (cutoff point = 2.5).

Variable	Total (n = 1393)	Non-IR group (n = 833)	IR group (n = 560)	*P* values
Age, yr	50.8 ± 11.1	50.5 ± 11.4	51.3 ± 10.6	.159
Male, n (%)	851 (61.1)	455 (54.6)	396 (70.7)	.000
Female, n (%)	542 (38.9)	378 (45.4)	164 (29.3)	.000
BMI (kg/m^2^)	25.62 ± 3.56	23.81 ± 3.17	226.82 ± 3.32	.000
SBP (mm Hg)	123.0 ± 17.9	120.9 ± 17.6	128.0 ± 17.7	.000
DBP (mm Hg)	75.7 ± 11.2	74.0 ± 11.0	78.2 ± 11.1	.000
FBG (mmol/L)	5.64 ± 1.29	5.32 ± 0.69	6.11 ± 1.74	.000
HbA1c (%)	5.87 ± 0.84	5.72 ± 0.58	6.10 ± 1.07	.000
Insulin (IU/mL)	10.81 ± 0.95	6.93 ± 2.16	16.57 ± 7.59	.000
TG (mmol/L)	1.49 (1.07, 2.19)	1.31 (0.96, 1.78)	1.59 (1.14, 2.46)	.000
TC (mmol/L)	5.14 ± 0.96	5.12 ± 0.94	5.16 ± 0.98	.416
LDL-C (mmol/L)	2.75 ± 0.75	2.72 ± 0.76	2.80 ± 0.73	.034
HDL-C (mmol/L)	1.55 ± 0.37	1.62 ± 0.38	1.46 ± 0.33*	.000
TSH (mIU/mL)	2.34 ± 1.03	2.41 ± 1.06	2.25 ± 1.00	.004
FT3 (pmol/L)	5.03 ± 0.71	4.96 ± 0.72	5.15 ± 0.66	.000
FT4 (pmol/L)	16.92 ± 2.32	17.05 ± 2.35	16.74 ± 2.26	.015
TFQI	0.00 (−0.28, 0.28)	0.03 (−0.54, 0.6)	−0.05 (−0.58, 048)	.000
TT4RI	35.14 (25.77, 49.58)	37.30 (27.01, 52.09)	32.36 (11.07, 53.65)	.000
TSHI	3.03 (2.68, 3.38)	3.09 (2.73, 3.44)	3.01 (2.68, 3.43)	.000
FT3/FT4	0.30 (0.27, 0.32)	0.29 (0.24, 0.33)	0.31 (0.25, 0.37)	.000
AIP	0.00 (−0.19, 0.21)	−0.1 (−0.27, 0.09)	0.15 (−0.21, 0.51)	.000
QUICKI	0.34 ± 0.03	0.36 ± 0.03	0.31 ± 0.02	.000

AIP = atherogenic index of plasma, BMI = body mass index, DBP = diastolic blood pressure, FBG = fasting blood glucose, FT3 = free triiodothyronine, FT4 = free thyroxine, HDL-C = high-density lipoprotein cholesterol, LDL-C = low-density lipoprotein cholesterol, PTFQI = Parametric Thyroid Feedback Quantile-based Index, QUICKI = quantitative insulin sensitivity check index, SBP = systolic blood pressure, TC = total cholesterol, TFQI = Thyroid Feedback Quantile-based Index, TG = triglycerides, TSH = thyroid-stimulating hormone, TSHI = Thyroid-Stimulating Hormone Index, TT4RI = Thyrotroph T4 Resistance Index.

* *P* < .05.

Among thyroid-related parameters, FT3 and the FT3/FT4 ratio were significantly higher in the IR group, whereas TSH, FT4, TFQI, TT4RI, and TSHI were all reduced (all *P* ≤ .015).

No significant differences were observed for age (*P* = .159) or total cholesterol (*P* = .416; Table 1).

### 3.2. Association of sensitivity to THs with HOMA-IR

We examined the relationship between thyroid function indices and IR using correlation regression analyses. Unadjusted Pearson correlations with continuous HOMA-IR were negligible for TFQI (*r* = −0.06, *P* = .019), TSHI (*r* = −0.06, *P* = .042), and TT4IR (*r* = −0.04, *P* = .137), while the FT3/FT4 ratio demonstrated a weak but statistically significant positive correlation (*r* = 0.217, *P* < .001; Table 2).

**Table 2:** Association between indices of thyroid hormone sensitivity and HOMA-IR.

Variables	*r*	*P* values
TFQI	−0.06	.019
TSHI	−0.06	.042
TT4IR	−0.04	.137
FT3/FT4	0.217	.000

FT3 = free triiodothyronine, FT4 = free thyroxine, HOMA-IR = Homeostatic Model Assessment for Insulin Resistance, OR = odds ratio, TFQI = Thyroid Feedback Quantile-based Index, TSHI = Thyroid-Stimulating Hormone Index, TT4RI = Thyrotroph T4 Resistance Index.

In multivariable logistic regression modeling, the binary outcome of HOMA-IR ≥ 2.5 (Table 3), independent associations were observed after mutual adjustment among thyroid indices. TFQI (OR = 0.85, 95% CI: 0.77–0.90), TSHI (OR = 0.80, 95% CI: 0.72–0.90), and TT4IR (OR = 0.82, 95% CI: 0.73–0.91) each emerged as significant protective factors (all *P* < .001). Conversely, the FT3/FT4 ratio independently predicted an increased risk of IR (OR = 1.50, 95% CI: 1.33–1.68, *P* < .001). These findings demonstrate that while linear correlations are weak, thyroid feedback integrity and the FT3/FT4 ratio exhibit robust, independent associations with IR risk (Table 3).

**Table 3:** Multivariable logistic regression (dependent: HOMA-IR ≥ 2.5).

Variables	*B*	OR (95% CI)	*P* values
TFQI	−0.271	0.85 (0.77–0.90)	.00
TSHI	−0.218	0.80 (0.72–0.90)	.00
TT4IR	−0.201	0.82 (0.73–0.91)	.00
FT3/FT4	0.42	1.50 (1.33–1.68)	.00

CI = confidence interval, FT3 = free triiodothyronine, FT4 = free thyroxine, HOMA-IR = Homeostatic Model Assessment for Insulin Resistance, OR = odds ratio, TFQI = Thyroid Feedback Quantile-based Index, TSHI = Thyroid-Stimulating Hormone Index, TT4RI = Thyrotroph T4 Resistance Index.

### 3.3. *Quartile analysis of thyroid hormone sensitivity indices and* IR *risk*

**Figure 2.**
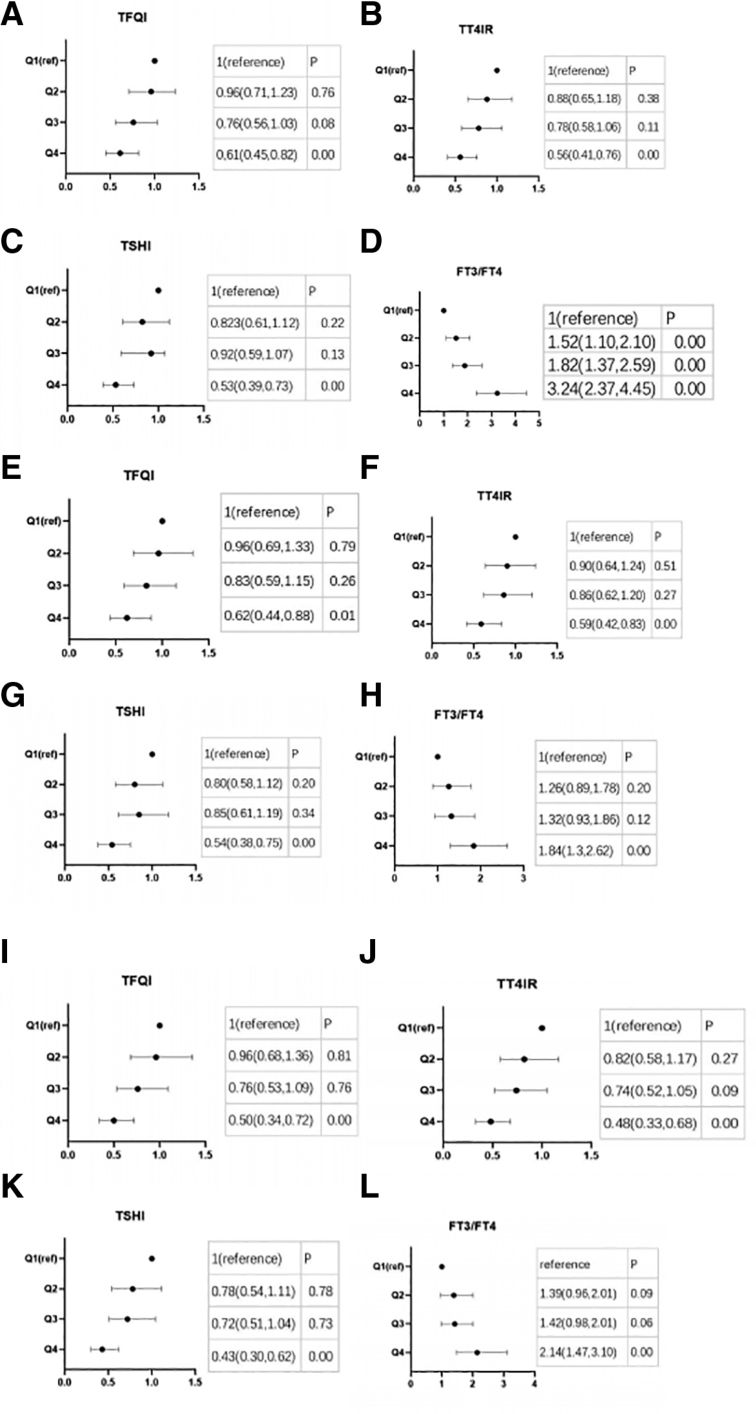
Quartile analysis of thyroid hormone sensitivity indices and insulin resistance risk. The forest maps of logistic regression analysis for the correlation between high IR level (dependent variable) and quartiles of thyroid hormone sensitivity indices (independent variables), with the first quartile serving as the reference – non-adjusted model (A) to (C); figures (D) to (F) were adjusted for the confounding factors, including sex, age, and BMI. Figures (G) to (H) were adjusted for the confounding factors, including sex, age, BMI, HbA1c, FBG, SBP, DBP, TG, and LDL-C. BMI = body mass index, DBP = diastolic blood pressure, FBG = fasting blood glucose, FT3 = free triiodothyronine, FT4 = free thyroxine, HbA1c = glycated hemoglobin, LDL-C = low-density lipoprotein cholesterol, SBP = systolic blood pressure, TFQI = Thyroid Feedback Quantile-based Index, TG = triglycerides, TSHI = Thyroid-Stimulating Hormone Index, TT4RI = Thyrotroph T4 Resistance Index.

The relationship between thyroid hormone sensitivity indices and IR using quartile-based logistic regression models is shown in Figure 2. In the unadjusted model, a clear gradient was observed for all indices. For TFQI, Q4 demonstrated a significantly reduced risk compared with Q1 (OR = 0.61, 95% CI: 0.45–0.82, *P* < .001), with incremental risk reduction across quartiles. Similarly, TT4RI Q4 exhibited markedly lower odds of IR (OR = 0.56, 95% CI: 0.41–0.76, *P* < .001), and TSHI Q4 showed the strongest protective effect (OR = 0.53, 95% CI: 0.39–0.73, *P* < .001; Fig. 2).

The FT3/FT4 demonstrated a dose-dependent positive association with IR risk. Compared with Q1, progressively higher quartiles showed increased odds: Q2 (OR = 1.52, 95% CI: 1.10–2.10), Q3 (OR = 1.82, 95% CI: 1.37–2.59), and Q4 (OR = 3.24, 95% CI: 2.37–4.45), all *P* < .001 (Fig. 2).

After multivariable adjustment, these associations persisted with attenuated magnitude. In the fully adjusted model, the protective effects remained statistically significant for Q4 of TFQI (OR = 0.50, 95% CI: 0.34–0.72, *P* < .001), TT4RI (OR = 0.48, 95% CI: 0.33–0.68, *P* < .001), and TSHI (OR = 0.43, 95% CI: 0.30–0.62, *P* < .001). For the FT3/FT4 ratio, the fully adjusted Q4 risk remained substantially elevated (OR = 2.14, 95% CI: 1.47–3.10, *P* < .001), though effect sizes decreased progressively with covariate adjustment. These findings demonstrate robust, independent dose–response relationships wherein greater thyroid hormone sensitivity is associated with reduced IR risk, while higher FT3/FT4 confers elevated risk across all models (Fig. 2).

### 3.4. Receiver operating characteristic curve for thyroid indices in predicting IR

**Figure 3.**
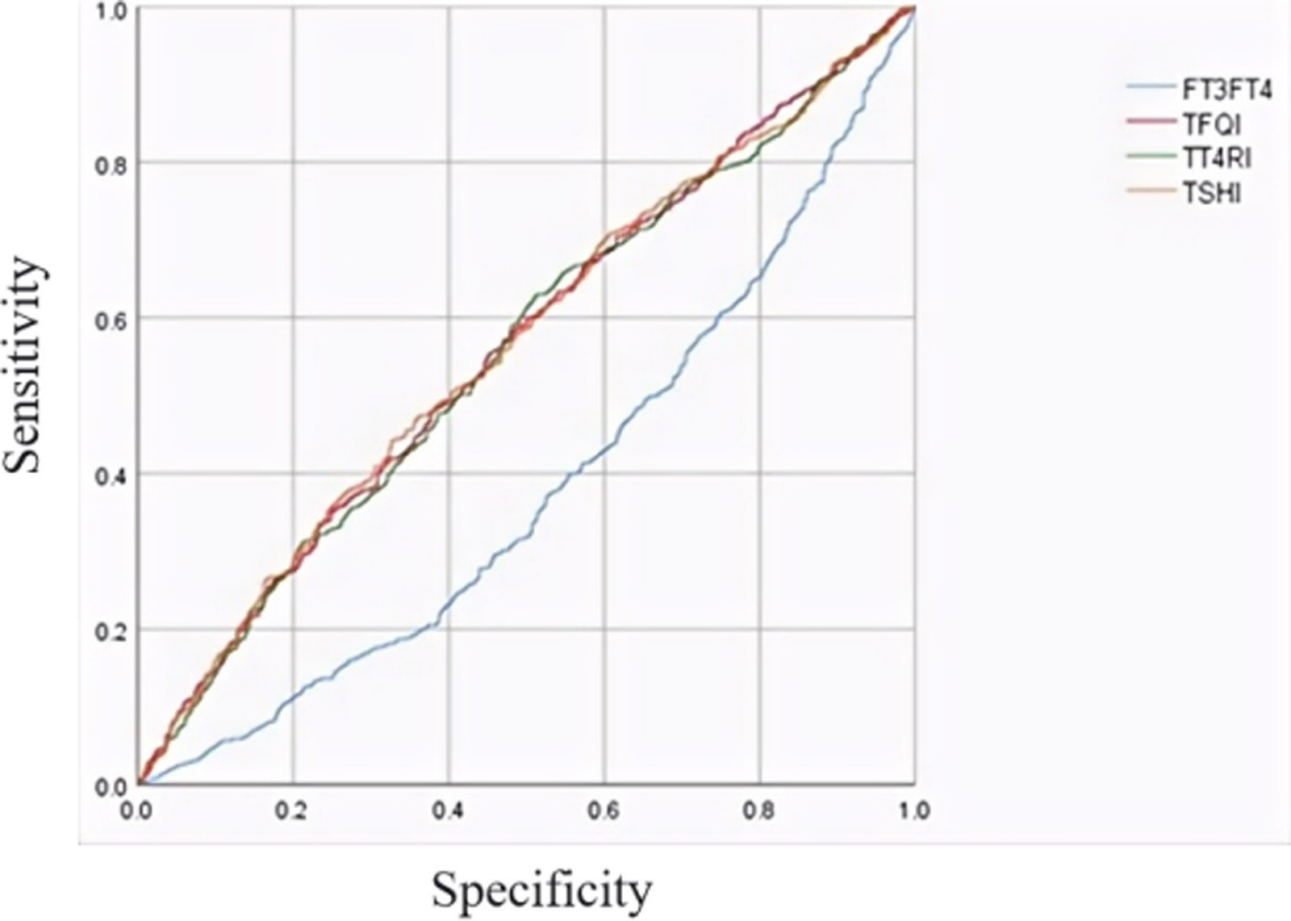
ROC curve for thyroid indices in predicting insulin resistance. FT3 = free triiodothyronine, FT4 = free thyroxine, ROC = receiver operating characteristic, TFQI = Thyroid Feedback Quantile-based Index, TSHI = Thyroid-Stimulating Hormone Index, TT4RI = Thyrotroph T4 Resistance Index.

The study used curve analysis to evaluate the discriminative accuracy of thyroid hormone sensitivity indices for predicting IR (HOMA-IR ≥ 2.5; Table 3). TFQI, TT4RI, and TSHI demonstrated modest but statistically significant discriminative ability, with areas under the curve (AUC) of 0.563, 0.557, and 0.564, respectively (all *P* < .001). These values indicate that each index correctly discriminates individuals with versus without IR approximately 56% of the time, performing slightly better than chance. The FT3/FT4 ratio exhibited paradoxical discrimination with an AUC of 0.383 (95% CI: 0.353–0.413, *P* < .001), significantly below the 0.50 reference line, suggesting that higher FT3/FT4 ratios are associated with a decreased probability of IR in this analysis (Table 4, Fig. 3).

**Table 4:** Discriminative accuracy of thyroid hormone sensitivity indices for insulin resistance.

Variables	Area	95% CI		*P* values
		Lower	Upper	
FT3/FT4	0.383	0.353	0.413	.000
TFQI	0.563	0.532	0.593	.000
TT4RI	0.557	0.527	0.588	.000
TSHI	0.564	0.534	0.595	.000

CI = confidence interval, FT3 = free triiodothyronine, FT4 = free thyroxine, TFQI = Thyroid Feedback Quantile-based Index, TSHI = Thyroid-Stimulating Hormone Index, TT4RI = Thyrotroph T4 Resistance Index.

## 4. Discussion

This cross-sectional study of 1393 euthyroid adults in Beijing revealed a significant inverse association between central thyroid hormone resistance (reflected by higher TFQI, TT4RI, and TSHI) and IR. Participants in the highest quartile of these indices had ~50% lower odds of IR after full HOMA-IR ≥2.5 adjustment for metabolic confounders. In contrast, the FT3/FT4 ratio, a marker of peripheral thyroid hormone conversion, was positively associated with IR. These associations followed a threshold effect, primarily observed in extreme quartiles, suggesting a nonlinear relationship.

This comprehensive cross-sectional study of 1393 euthyroid adults provides compelling evidence for a significant inverse association between central resistance to THs and IR. The core of our findings lies in the consistent signal from 3 distinct indices of pituitary-hypothalamic sensitivity: TFQI, TT4RI, and TSHI. Participants in the highest quartile (Q4) of these indices exhibited an approximately 50% reduction in the odds of having HOMA-IR ≥2.5, even after rigorous adjustment for a comprehensive panel of confounders, including age, sex, BMI, BP, HbA1c, fasting glucose, and lipids. This protective association was not linear but demonstrated a clear threshold effect, becoming statistically significant only in the highest quartile. This pattern suggests the existence of a distinct neuroendocrine phenotype rather than a gradual metabolic shift.

Conversely, we observed a robust positive association between the FT3/FT4 ratio, a widely recognized surrogate for peripheral thyroxine to triiodothyronine conversion (primarily driven by deiodinase type 1 and 2 activity), and HOMA-IR.^[14,16]^ This diametric opposition, where central resistance appears protective and peripheral activation detrimental, paints a more nuanced picture of thyroid hormone action in metabolism, moving beyond the simplistic “more hormone equals higher metabolism” paradigm.

Our results both confirm and refine the growing body of evidence linking thyroid homeostasis to metabolic health. Previous large-scale studies, such as those by Laclaustra et al and Zhou et al, established that impaired thyroid hormone sensitivity is a risk factor for metabolic syndrome and its components.^[10,11,17]^ Our work narrows this focus specifically to HOMA-IR quantified IR within the euthyroid Chinese population, thereby minimizing confounding by overt thyroid dysfunction.

The seemingly paradoxical protective effect of central resistance finds precedent in subtle clues from earlier research. For instance, population studies have occasionally noted that individuals with “high-normal” FT4 levels, who would be expected to have fully suppressed TSH if the feedback loop were hypersensitive, sometimes exhibit more favorable metabolic profiles. Our indices, particularly the TFQI, mathematically encapsulate this exact “high-normal FT4 with non-suppressed TSH” phenotype.^[14]^ This biochemical signature suggests a resetting of the hypothalamic–pituitary–thyroid (HPT) axis set point, which may be an adaptive, compensatory response to underlying metabolic stressors, ultimately conferring a degree of insulin sensitivity.

The strong positive correlation between the FT3/FT4 ratio and HOMA-IR is a critical piece of the puzzle. This finding supports the hypothesis that increased peripheral conversion of T4 to the more biologically active T3 may be a double-edged sword. While essential for normal physiology, excessive conversion could be driven by chronic hyperinsulinemia, as insulin is a known stimulator of hepatic deiodinase type 1 (D1) activity.^[10]^ This creates a potential vicious cycle: IR → hyperinsulinemia → increased T3 production → heightened catabolic state and lipolysis → increased free fatty acid flux → worsened peripheral IR.^[18,19]^ Our data are consistent with this model, positioning the FT3/FT4 ratio not just as a marker of thyroid function but potentially as an indirect reflector of chronic insulin tone.^[17,20]^

### 4.1. Delineation of potential pathophysiological mechanisms

The basis for our observations can be theorized across several interrelated pathways: the central set-point hypothesis: we propose that a mildly resistant central HPT axis allows for a slightly higher circulating FT4 level without triggering appropriate TSH suppression. This elevated FT4 “window” within the strict euthyroid range may enhance metabolic efficiency in peripheral tissues. For example, in the liver, FT4 can activate AMP-activated protein kinase, a master regulator of energy homeostasis, leading to suppressed gluconeogenic genes and improved lipid oxidation.^[21,22]^ In skeletal muscle, even a minor uptick in thyroid hormone tone could augment GLUT-4 translocation and mitochondrial oxidative phosphorylation, thereby improving glucose disposal.^[23]^ The lipid-mediated improvement pathway: THs are potent regulators of lipid metabolism. The higher FT4 levels associated with a resistant central axis likely promote LDL receptor expression and lipoprotein lipase activity. This is corroborated by our data, which show a more favorable TG and HDL-C profile in the high-resistance quartiles (data not shown). Improved lipid profiles directly reduce lipotoxicity, a key driver of IR in muscle and liver.^[11]^ The peripheral overdrive hypothesis: the positive link between the FT3/FT4 ratio and IR suggests that in some individuals, excessive peripheral generation of T3 may be detrimental. Elevated T3 can increase basal metabolic rate and sympathetic nervous system activity, promoting lipolysis and gluconeogenesis.^[24,25]^ The resulting influx of free fatty acids and glycerol to the liver provides substrate for ectopic fat deposition and increased glucose production, directly antagonizing insulin action.^[11]^ Inflammatory pathways: beyond traditional metabolic pathways, emerging evidence implicates immune modulation. TSH has been shown to directly act on macrophages via the thyroid stimulating hormone receptor,^[26]^ promoting M1 polarization and the secretion of pro-inflammatory cytokines like interleukin-1α/β and interleukin-6, which can upregulate the EGR1-LCN2/SOCS3 signaling axis and inhibit IRS1-AKT insulin signaling. This represents a direct, thyroid hormone-independent pathway through which elevated TSH (a component of the central resistance indices) could exacerbate IR, contrasting with the potential compensatory benefits of the overall central resistant phenotype observed in our study.

Our findings introduce a potentially valuable tool for metabolic risk stratification. The indices TFQI, TT4RI, and TSHI are derived from routine, low-cost tests (TSH and FT4) and could be automatically calculated and reported by laboratory information systems. Their modest but significant power for IR (AUC ~0.68) suggests they may add incremental value to current diabetes prediction models.

Our data sound a note of caution regarding the long-term metabolic consequences of aggressive TSH-suppressive therapy with levothyroxine in patients with thyroid cancer or benign nodules, suggesting a need to balance oncological benefits against potential metabolic risks.

## 5. Limitations

Several limitations in this study should be acknowledged. First, our research data are only from our hospital. The conclusion needs to be verified in a larger population. Second, as with other observational designs, the causal relationship might not be inferred, and potential residual confounding was unavoidable, although we have adjusted for the major confounders. Third, the cross-sectional nature is the most significant constraint, as it prevents us from establishing causality. Future prospective cohort studies are warranted to determine whether thyroid hormone sensitivity is an independent risk factor for the incidence of type 2 diabetes and coronary heart disease in euthyroid individuals.

## 6. Conclusion

In conclusion, our findings demonstrate a paradoxical dissociation within the thyroid–insulin axis in euthyroid adults. Reduced central sensitivity to THs, reflected by elevated TFQI, TT4RI, and TSHI, is independently associated with a lower prevalence of IR. Conversely, an elevated FT3/FT4 ratio, suggestive of enhanced peripheral deiodination, is linked to a more adverse metabolic phenotype characterized by greater IR. This compelling dichotomy underscores that the metabolic consequences of thyroid function are not merely a function of hormone concentrations but are critically determined by the allostatic set-point of the HPT axis and the rate of peripheral hormone activation. The assessment of these dynamics via calculable indices provides a novel framework for risk stratification in the metabolically heterogeneous, yet biochemically euthyroid, general population.

## Acknowledgments

We thank the staff and the participants of the study for their valuable contributions. We are especially grateful to Professor Su Fenqi of the Department of Statistics, Hebei Medical University, for his expert guidance and invaluable contributions to the statistical analyses in this work.

## Author contributions

**Conceptualization:** Lintao Shi.

**Resources:** Min Zhang.

**Methodology:** Aiyun Yu.

**Software:** Aiyun Yu, Lijun Li.

**Investigation:** Weihan Zhang, Jianjun Wang, Lijun Li.

**Supervision:** Lijun Li.

**Writing – original draft:** Lintao Shi, Min Zhang.

**Writing – review & editing:** Lintao Shi, Weihan Zhang, Jianjun Wang, Lijun Li.
